# Time-Series Transcriptome of *Cucumis melo* Reveals Extensive Transcriptomic Differences with Different Maturity

**DOI:** 10.3390/genes15020149

**Published:** 2024-01-24

**Authors:** Fengjuan Liu, Xupeng Shao, Yingying Fan, Binxin Jia, Weizhong He, Yan Wang, Fengzhong Wang, Cheng Wang

**Affiliations:** 1 Institute of Quality Standards & Testing Technology for Agro-Products, Xinjiang Academy of Agricultural Sciences, Urumqi 830091, China; liufengjuan@xaas.ac.cn (F.L.); shaoxupeng@xaas.ac.cn (X.S.); fyyxaas@xaas.ac.cn (Y.F.); jiabinxin@xaas.ac.cn (B.J.); hewei198112@xaas.ac.cn (W.H.); wangyan@xaas.ac.cn (Y.W.); 2 Key Laboratory of Agro-Products Quality and Safety of Xinjiang, Laboratory of Quality and Safety Risk Assessment for Agro-Products (Urumqi), Ministry of Agriculture and Rural Affairs, Key Laboratory of Functional Nutrition and Health of Characteristic Agricultural Products in Desert Oasis Ecological Region (Co-Construction by Ministry and Province), Ministry of Agriculture and Rural Affairs, Urumqi 830091, China; 3 Key Laboratory of Agro-Products Quality and Safety Control in Storage and Transport Process, Ministry of Agriculture and Rural Affairs, Institute of Food Science and Technology, Chinese Academy of Agricultural Sciences, Beijing 100193, China

**Keywords:** *Cucumis melo*, fruit ripening, different maturity, climacteric fruits, transcriptome

## Abstract

As the most important melon cultivar grown in the north-western provinces of China, Hami melon (*Cucumis melo*) produces large edible fruits that serve as an important dietary component in the world. In general, as a climacteric plant, melon harvested at 60% maturity results in a product with bad quality, while the highest-quality product can be guaranteed when harvesting at 90% maturity. In order to clarify the genetic basis of their distinct profiles of metabolite accumulation, we performed systematic transcriptome analyses between 60% and 90% maturity melons. A total of 36 samples were sequenced and over 1.7 billion reads were generated. Differentially expressed genes in 60% and 90% maturity melons were detected. Hundreds of these genes were functionally enriched in the sucrose and citric acid accumulation process of *C. melo*. We also detected a number of distinct splicing events between 60% and 90% maturity melons. Many genes associated with sucrose and citric acid accumulation displayed as differentially expressed or differentially spliced between different degrees of maturity of Hami melons, including *CmCIN2*, *CmSPS2*, *CmBGAL3*, and *CmSPS2*. These results demonstrate that the phenotype pattern differences between 60% and 90% maturity melons may be largely resulted from the significant transcriptome regulation.

## 1. Introduction

Melon (*C. melo* L.), a member of the Cucurbitaceae family, is an economically important fruit crop widely cultivated in the world [1]. According to FAO, the annual yield of melon is estimated to be around 30 million tons in the world, in which more than half of the global production is contributed by China (http://www.fao.org, accessed on 2 April 2023). It is especially important for countries in the Mediterranean and East Asian regions, from which a significant and growing economic value has come from hybrid varieties [1]. Earlier studies have proposed that melon originated in Africa. However, more recent data suggest that melon originated in Asia [2]. Melon has an extensive polymorphism and has been divided into at least 19 botanical groups [3]. With high variability in phenotypic characteristics, melon has attracted much attention to trace the precursors of modern genetics [4]. Considering its economic importance and specific biological properties, melon has become an attractive model for studying the genetic basis of valuable biological characteristics, including sex determination [5,6], phloem physiology [7], and fruit ripening [8].

Based on the type of fruit ripening, fruit-bearing plants can be broadly classified into two groups: climacteric or non-climacteric [9]. Climacteric fruits (e.g., apple, banana, tomato, and avocado) are characterized by a sudden rise in respiration followed by the autocatalytic synthesis of ethylene, a strong aroma, orange pulp, ripening abscission, and a short shelf life with a rapid loss of firmness and taste deterioration [10]. By contrast, non-climacteric fruits are characterized by a limited amount of ethylene synthesis in the mature product (e.g., grape, *Citrus* spp., strawberry, and pineapple), white pulp, low aroma, no ripening abscission, and a longer shelf life [8,11,12]. Fruit ripening has a strong influence on many organoleptic properties of the fruit, including color, sweetness, aroma, flavor, acidity, and firmness [10,13]. For most climacteric fruits, the quality generally deteriorates quickly after maturity, which severely limits the potential market available to commercial fruit producers [11]. Therefore, strict control on harvest time has a significant impact on the economic value of climacteric fruits (e.g., melon in this study).

The fruit ripening process of melon typically contains four main stages: young fruit, expanding, premature, and mature. Ten days after pollination (10 DAP), ovarian epidermal cells stop to proliferate and lead the young fruit stage to become a small volume of fruit. Afterwards, the cell diameters of the mesocarp, endocarp, and placenta grow rapidly, resulting in fruit enlargement. Twenty days after pollination (20 DAP), the fruit is in an expanding stage, the fruit volume is increased, and the sugar content is very low. Then, the fruit length grows continuously, accompanied by the sucrose content of the fruit increasing quickly. Thirty days after pollination (30 DAP), the fruit of melon in the premature stage, with stopped cell expansion, maximum fruit volume, and high flesh firmness, is obtained. During this stage, the sucrose content increases continuously until the maximum content (60% of total sugar) is reached and the flesh turns soft in texture. Forty-five days after pollination (45 DAP), the fruit of melon is thought to be fully mature, with the sucrose content and other nutritional components increasing to the highest level.

In recent years, there has been a prevailing trend among farmers to harvest melons at 60–70% maturity, aiming for early market entry and enhanced pricing. However, this practice has adversely impacted the quality and flavor of melons. Given the climacteric nature of melon plants, optimal quality is attained at 90% maturity. The precise mechanisms underlying the undesired effects of harvesting at 60% maturity remain unclear. To address this, our study focuses on the “Xizhoumi 25” Hami melon, the most cultivated cultivar in Xinjiang, as a model to explore the genetic intricacies contributing to low-quality melons resulting from early harvesting. Sampling melons at both 60% and 90% maturity, we mimicked commercial storage conditions, utilizing room temperature and specific humidity levels. High-throughput sequencing was employed to compare the transcriptome of melons at these maturity stages, providing insights into the molecular processes influencing melon quality variations post-harvest.

## 2. Materials and Methods

### 2.1. Plant Material

“Xizhoumi 25” Hami melon plants were grown in the greenhouse of Xinjiang University. Flowers of the plant were tagged at anthesis to record the number of days after pollination. The fruits were collected at two different development stages, 33 and 42 DAP, and then stored at room temperature (25 °C). The mesocarp of the melon on day 0, 3, 5, 7, 14, and 21 was sampled and frozen immediately in liquid nitrogen and stored at −80 °C until analyses. For each time-point, three fruits were harvested as biological replicates from three different plants.

### 2.2. Measurement of the Quality of Melon Flesh

We used an FT 011 fruit firmness tester to determine the center firmness of the flesh using the fruit cut lengthwise. After homogenizing the center flesh, the total soluble solids content (TSS, Brix%) was measured using a laboratory refractometer (HC-112ATC, Shanghai LICHENKEYI, Shanghai, China) and PHB-4 (Shanghai LICHENKEYI, Shanghai, China) equipment. The respiratory intensity (unit: CO_2_ mg/(kg·h)) was measured using standing lye absorption methods as recently described [14]. The content of sugars (fructose, glucose, and sucrose) and organic acids (malic acid, citric acid, and succinic acid) in the fruit flesh were measured using high-performance liquid chromatography (Waters 2695, Waters, Milford, MA, USA) using the following methods. The organic acid composition was analyzed in 10 μL aliquots of a 1:10 juice solution using a non-polar derivative Lichrospher column (RP-Select B, 5 μm; Merck). The mobile phase (0.3 mL min^−1^) was a 99:1 *v/v* combination of deionized water (as described above) and methanol with a buffer of 50 mM phosphate di-hydrogen potassium with H_2_SO_4_ (pH 3), previously filtered with 45 μm cellulose (Albet, Vic, Barcelona, Spain) and degassed with an ultrasonic system (model Ultrasons; Selecta S.A., Abrera, Spain). Samples were read at 210 nm with a UV-vis detector (model UV-2700; SHIMADZU) for 45 min at 30 °C.

### 2.3. RNA Extraction and Sequencing

The total RNA from each sample group was extracted by using a TRIZOL reagent (Invitrogen, Carlsbad, CA, USA). The extracted RNA was further purified using an RNeasy mini kit (QIAGEN, Germantown, MD, USA). The degradation and contamination of total RNA were tested before the RNA library was prepared with 1% agarose gels. The RNA integrity was evaluated using a Qubit 2.0 Fluorometer and 2100 Bioanalyzer (Agilent Technologies, Santa Clara, CA, USA). The qualified RNA from each sample was used to construct the sequencing library using an Illumina TruSeq Stranded RNA Kit (Illumina, San Diego, CA, USA) following the manufacturer’s instructions. The purified cDNA libraries were enriched using PCR. After cluster generation, all the libraries were sequenced on an Illumina HiSeq 2500 platform with paired-end 150 bp reads (Novogene Bioinformatics Institute, Beijing, China). Together, a total of 36 cDNA libraries were sequenced in this study.

### 2.4. Differentially Expressed Gene Identification

Raw sequencing reads for each sample were examined for quality using FastQC (v 0.11.3) (https://www.bioinformatics.babraham.ac.uk/projects/fastqc/, accessed on 2 February 2023) with default settings. Low-quality reads were removed using fastp (v 0.19.5) [15] with default settings. High-quality reads of each sample were mapped to the melon reference genome (*C. melo* L.) [1] using Hisat2 (v 2.1.0) [16]. The gene expression level (FPKM, fragments per kilobase million) was calculated using the RSEM program (v 1.3.1) [17]. The raw read counts for each gene estimated by RSEM were extracted and normalized using TMM to control the differences in sequencing depth among samples. Differentially expressed genes were detected using DESeq2 (v 1.10.1) [18] with a minimal fold change of 2 and an adjusted *p*-value cutoff of 0.05.

### 2.5. Characterization of Alternative Splicing Events

Five basic types of alternative splicing events (ASEs) have been studied in this article, including skipped exon (SE), mutually exclusive exions (MXE), alternative 5′ splice site (A5SS), alternative 3′ splice site (A3SS), and retained intron (RI) [19]. rMATS [20] was firstly used to identify and count the reads that correspond to each of the five types of ASEs. It identifies these ASEs events from a GTF file of annotated transcripts and detects the number of reads that correspond to each of the five events described. To identify differential alternative splicing (DAS) events between 60% and 90% maturity melons, an FDR-adjusted *p*-value 0.05 was used as a threshold.

### 2.6. Enrichment Analysis of Differentially Expressed Genes and Alternative Splicing (AS) Analysis

Overrepresentation of the gene ontology (GO) terms for the differentially expressed genes (DEGs) was determined using the Goenrich webserver (http://cucurbitgenomics.org/goenrich, accessed on 22 April 2023). Dataset melon (DHL92) (v3.61) was used in this analysis. Alternative splicing is an important mechanism for regulating the expression of genes and the variables of proteins. rMATS (3.2.5) software was used to analyze the AS event. The cutoff *p*-value was set to 0.05. Protein–protein interactions for the DEGs with experimental or database evidence were retrieved from the STRING database [21].

## 3. Results

### 3.1. Rapid Changes in Fruit Firmness and Metabolic Profiles between 60% and 90% Maturity

**Figure 1:**
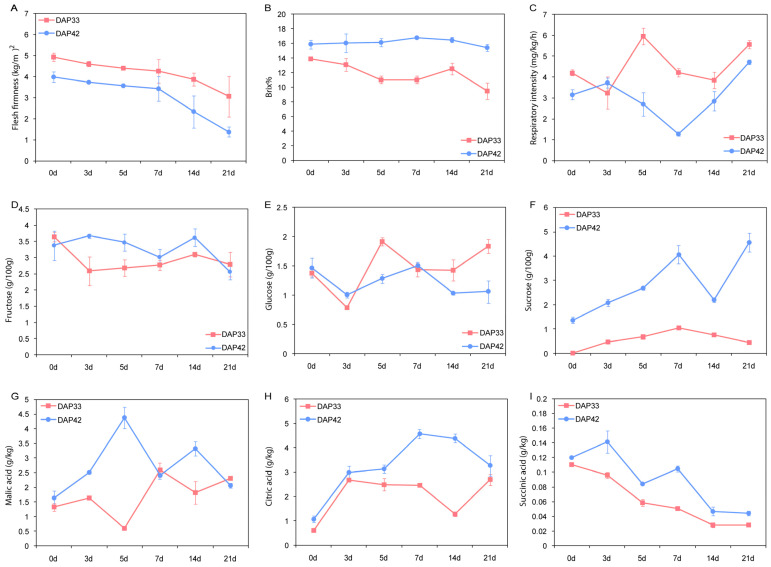
Comparison of 60%(DAP33) and 90% (DAP42)maturity melon in flesh firmness (**A**), total soluble solids content (**B**), respiratory intensity (**C**), fructose (**D**), glucose (**E**), sucrose (**F**), malic acid (**G**), citric acid (**H**), and succinic acid (**I**) during storage.

Melon samples were taken from two different growth stages (33 and 42 DAP) which represent 60% and 90% maturity. These samples were stored at room temperature (25 °C) until use. We found that there are distinct pattern differences of the phenotype between 60% and 90% maturity melons. Specifically, the firmness of fruits in the 33 DAP group was higher than that in the 42 DAP group. The firmness of fruits from both groups gradually declined during storage (Figure 1A). The TSS of samples from the 42 DAP group reached a plateau at seven days of storage and remained relatively stable during storage. However, the TSS of samples from the 33 DAP group gradually decreased during the 21-day storage, and it was lower than that of the 45 DAP group in all of the testing points (Figure 1B). In contrast, the respiratory intensity of 33 DAP was stronger than that of the 42 DAP group. A large fluctuation in both groups can be observed in the respiratory intensity results (Figure 1C).

The contents of soluble sugar, including fructose, glucose, and sucrose, in the fruit flesh are largely related to product quality. The fructose and glucose contents were measured with high-performance liquid chromatography (HPLC). As shown in Figure 1D,E, similar patterns were obtained in the 33 DAP group and 42 DAP group during storage. However, the sucrose content of 42 DAP was significantly higher than that of the 33 DAP group. A two-fold increase in the sucrose content of 42 DAP was observed after 21 days of storage (Figure 1F). These results indicate that a rapid accumulation of sucrose in the 90% maturity melon contributed significantly to the sweet taste of the fruit flesh.

The contents of malic acid, citric acid, and succinic acid were detected using HPLC. Overall, the contents of malic acid, citric acid, and succinic acid were higher in the 42 DAP group. The contents of malic acid and citric acid increased gradually in line with increased storage time (Figure 1G,H), while the content of succinic acid decreased (Figure 1I). These results suggest that the rapid accumulation of citric acid during storage may be related to the sour flavor of the fruit flesh. In summary, significant differences of the phenotype between 60% and 90% maturity melons, which may largely contribute to the sweet taste and product quality, were observed in these tests.

### 3.2. Transcriptome Profiles of Storage between 60% and 90% Melon Maturity

In order to investigate the potential genetics basis underlying the distinct profiles of metabolite accumulation between 60% and 90% maturity melons, we performed RNA-sequencing analysis on the fruit flesh to generate their transcriptome profiles. In total, 36 libraries were sequenced and analyzed. After filtering out the low-quality reads, the average number of reads per sample was 47.9 million (Table S1). The RNA-Seq reads were mapped to the reference genome of melon using Hisat2 (v 2.1.0) [16]. The majority of reads from each sample (average 80.79%) were successfully mapped to the melon reference genome, suggesting a high quality of the sequenced reads for each sample. Between 27 and 57 million reads per sample were uniquely aligned to the melon genome (Table S1) and over 15,533 expressed genes were detected in each sample (Table S2).

**Figure 2:**
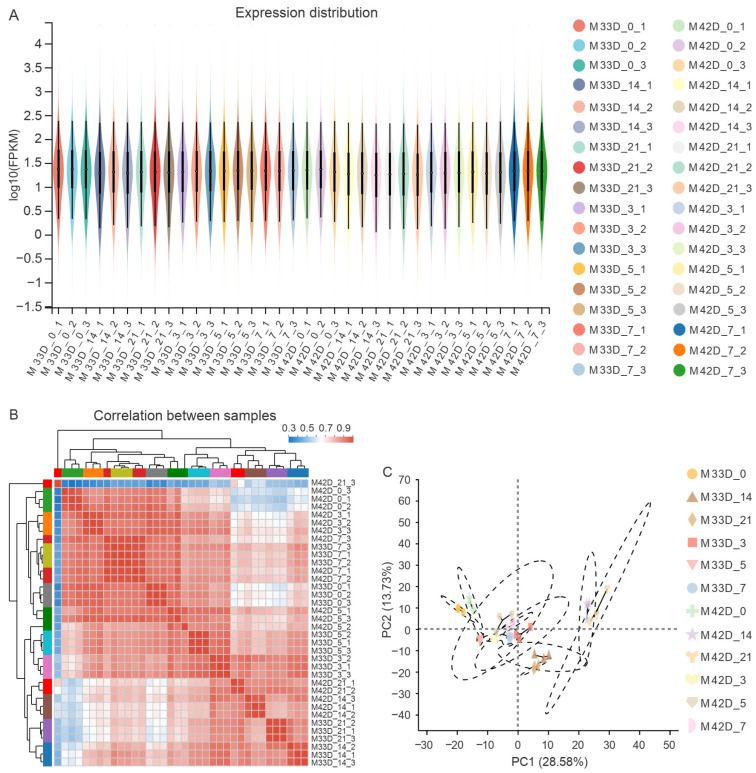
An overview of the transcriptome sequencing. (**A**) Distribution of expression levels for all melon genes across each sample. (**B**) Hierarchical clustering analysis of gene expression levels in each of the 36 samples. (**C**) PCA analysis of clustering of the 36 samples.

We estimated the expression level of each gene using FPKM. Approximately 47.91% (14,363 out of the 29,980 melon genes) of the expressed genes were in the 0–1 FPKM range, and 10.93% (3278 out of the 29,980 melon genes) of the expressed genes showed very high levels of expression (higher than 60 FPKM) (Figure 2A and Table S3). Genes were removed from subsequent analysis when normalized reads were lower than 1 FPKM. We compared the gene expression levels among different experimental groups. The expression patterns among biologically replicates were highly related (Figure 2B,C) and the correlation coefficient was close to 1 for replicates. On the whole, the high-quality RNA-Seq datasets generated in this study provide a solid base for identifying key genes participating in the distinct profiles of metabolite accumulation between 60% and 90% maturity melons.

### 3.3. Differentially Expressed Genes between 60% and 90% Maturity Melons

**Figure 3:**
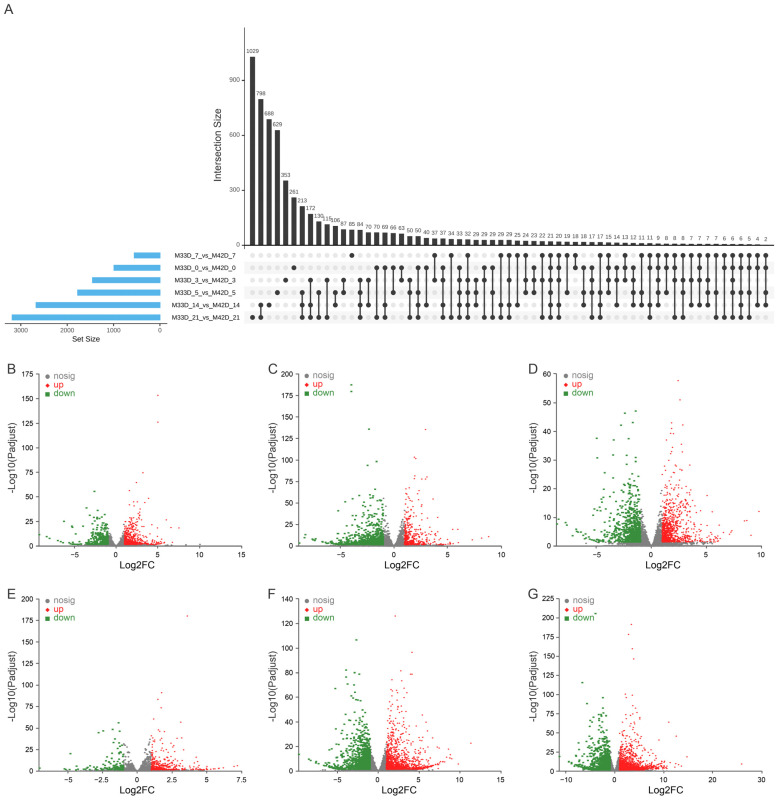
Differentially expressed genes between 60% and 90% melon maturity. (**A**) Upset plot between the 6 groups of differentially expressed genes. (**B**) Differentially expressed in 0 day of storage between 60% and 90% melon maturity. (**C**) Differentially expressed in 3 day of storage between 60% and 90% melon maturity. (**D**) Differentially expressed in 5 day of storage between 60% and 90% melon maturity. (**E**) Differentially expressed in 7 day of storage between 60% and 90% melon maturity. (**F**) Differentially expressed in 14 day of storage between 60% and 90% melon maturity. (**G**) Differentially expressed in 21 day of storage between 60% and 90% melon maturity.

We performed a pairwise comparison at six time-points of storage between 60% and 90% melon maturity to identify the genes underlying their distinct profiles of metabolite accumulation. We detected a total of 10,624 genes showing significant up-regulation or down-regulation (adjusted *p*-value < 0.05, fold change > 2 or < 0.5) during fruit storage (Figure 3A and Table S4). Of these, 990 were differentially expressed in 0 days of storage between 60% and 90% melon maturity, with 648 up-regulated and 342 down-regulated (Figure 3B); 1453 were differentially expressed in 3 days of storage between 60% and 90% melon maturity, with 466 up-regulated and 987 down-regulated (Figure 3C); 1773 were differentially expressed in 5 days of storage between 60% and 90% melon maturity, with 915 up-regulated and 858 down-regulated (Figure 3D); 552 were differentially expressed in 7 days of storage between 60% and 90% melon maturity, with 395 up-regulated and 157 down-regulated (Figure 3E); 2670 were differentially expressed in 14 days of storage between 60% and 90% melon maturity, with 1311 up-regulated and 1359 down-regulated (Figure 3F); and 3186 were differentially expressed in 21 days of storage between 60% and 90% melon maturity, with 1719 up-regulated and 1467 down-regulated (Figure 3G). Overall, many more genes showed differential expression at different storage time-points between 60% and 90% maturity melons, suggesting that much more dynamic transcriptome regulation happened between 60% and 90% maturity melons. This result is in agreement with the fact that more phenotypic divergence happened during storage between 60% and 90% melon maturity (Figure 1 and Table 1).

In order to gain further insights into the biologic function that differed between 60% and 90% maturity melons, we performed gene ontology (GO) enrichment analysis using FDR (false discovery rate). An adjusted *p*-value < 0.05 on DEGs was used to characterize the differences of storage between 60% and 90% maturity melons. Additional file 5: Table S5 lists the assigning of GO terms according to the molecular function (MF), biological process (BP), and cellular component (CC). We found that genes related to various biological processes, including metabolic, physiological, transport, and ion binding, were highly enriched in the up-regulated genes in 90% maturity melon. By contrast, the up-regulated genes in 60% melon maturity were highly enriched in GO categories, including catalytic activity and membrane localization. Taken together, these results indicate that there are significant differences in the up-regulated genes between 60% and 90% maturity melons (Figure S1).

### 3.4. Alternative Splicing between 60% and 90% Melon Maturity

**Figure 4:**
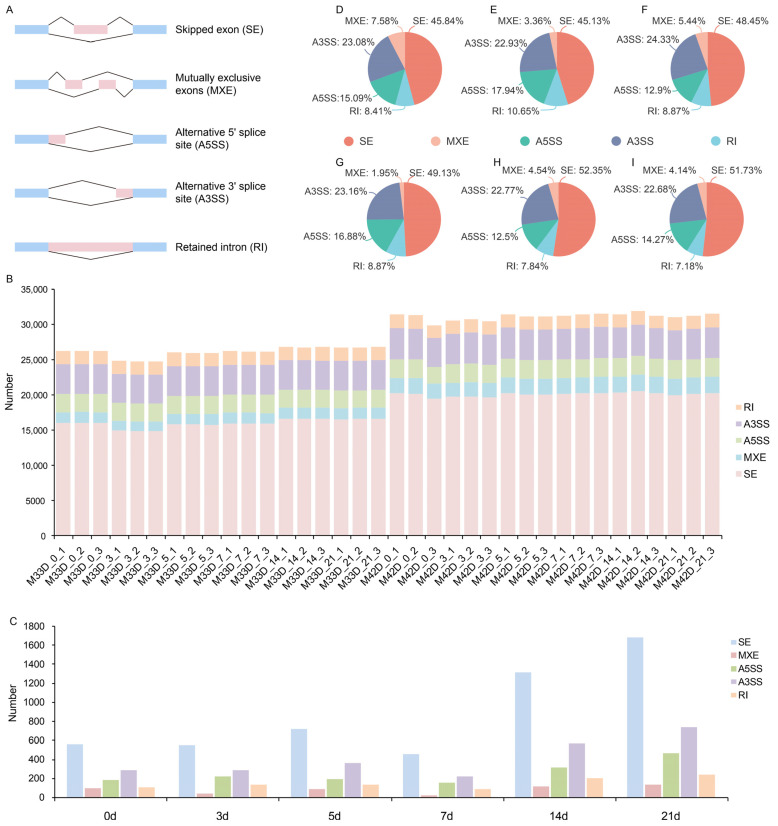
Alternative splicing events (ASEs) between 60% and 90% melon maturity. (**A**) Schematic representation of five types of alternative splicing events. Alternative exons are shown as pink boxes and flanking constitutive exons are shown as light blue boxes. (**B**) Distribution of isoform numbers for genes in melon genome. (**C**) Significantly differential ASEs between 60% and 90% melon maturity. D-I. Pie chart showing the percentage distribution of ASEs between 60% and 90% melon maturity in 0 (**D**), 3 (**E**), 5 (**F**), 7 (**G**), 14 (**H**), and 21 (**I**) day of storage.

As an important mechanism of posttranscriptional modification, alternative splicing (AS) can regulate multiple physiological processes in plants, including fruit ripening. To examine the influence of alternative splicing regulation in storage between 60% and 90% melon maturity, alternative splicing events (ASEs), including skipped exon (SE), mutually exclusive exons (MXE), alternative 5′ splice site (A5SS), alternative 3′ splice site (A3SS), and retained intron (RI) (Figure 4A), were first identified using rMATS [20].

We found that, on average, more than 28,000 genes (from 24,699 to 31,871) of the melon genome have alternative splicing events in the 36 transcriptomes (Figure 4B). Generally, many more alternative splicing events were found between 60% and 90% melon maturity with increased storage time, which is consistent with the number of DEGs identified. The most abundant ASEs were skipped exon, accounting for 60% of all ASEs, followed by alternative 3′ splice site (16%), alternative 5′ splice site (10%), retained intron (7%), and mutually exclusive exons (5%) (Figure 4B). With a *p*-value cutoff of 0.05, differential ASEs were identified between 60% and 90% melon maturity at the six time-points of storage (Table S6).

We identified a total of 10,617 genes showing significantly alternative splicing during fruit storage between 60% and 90% melon maturity (Figure 4C and Table S6). Among these, 1213 were significantly alternative spliced in 0 days of storage between 60% and 90% melon maturity; 1221 were significantly alternative spliced in 3 days of storage between 60% and 90% melon maturity; 1488 were significantly alternative spliced in 5 days of storage between 60% and 90% melon maturity; 924 were significantly alternative spliced in 7 days of storage between 60% and 90% melon maturity; 2512 were significantly alternative spliced in 14 days of storage between 60% and 90% melon maturity; and 3259 were significantly alternative spliced in 21 days of storage between 60% and 90% melon maturity. On the whole, many more genes showed significantly alternative splicing between 60% and 90% melon maturity, which is consistent with the fact that many more genes showed differential expression with increased storage time. Among these significantly alternative splicing events, the most abundant ASEs were skipped exon for all six time-points of storage (Figure 4D–I). This result is also in agreement with the fact that more phenotypic divergence happened between 60% and 90% melon maturity (Figure 1).

## 4. Discussion

The ripening and development of fruit represent intricately regulated processes that unfold through genetically programmed and irreversible mechanisms. These processes encompass a spectrum of physiological, biochemical, and organoleptic changes, exerting profound effects on the overall quality of the fruit, including attributes such as flavor, texture, color, and aroma [22]. It is noteworthy that harvesting fruits at an earlier growth stage can result in the deterioration of fruit quality, a phenomenon observed even in climacteric plants. This underscores the critical importance of understanding the molecular intricacies governing fruit development and ripening. In recent years, several studies have endeavored to unravel the molecular basis of these processes, particularly focusing on aspects such as fruit and peel color during development. Utilizing both transcriptomic and metabolic data, these investigations have contributed valuable insights into the underlying genetic and biochemical mechanisms steering the quality attributes of fruits [23,24,25]. This multidimensional approach offers a comprehensive understanding of the intricate interplay between genetic factors and metabolic processes that define the final quality characteristics of harvested fruits.

In this study, we employed an important commercial climacteric melon fruit, Hami melon (*C. melo*), as experimental material to investigate the genetic basis underlying the distinct profiles of metabolite accumulation between 60% and 90% melon maturity. Phenotypic differences between 60% and 90% melon maturity have been quantified. The results showed that there are significant differences in the firmness of the fruit, TSS content, respiratory intensity, fructose content, glucose content, sucrose content, malic acid content, citric acid content, and succinic acid content between these two maturity groups. Melons are mostly consumed because of their sweet taste [13], which is typically characterized by the accumulation of sucrose when the fruit development reaches the late stage [26,27,28,29,30]. Our results showed that the sucrose content was significantly higher in 90% maturity melon than that in 60% maturity melon, and it increased significantly in the 90% maturity melon group during storage. In melons with the desired sweet taste, besides sucrose as the primary component determining fruit quality [13,26], the content of organic acid can also affect the taste of melons [31,32,33,34]. By combining the high sugar and high acidity traits of melons, breeders have developed the “Xizhoumi 25” Hami melon. Our results showed that the contents of organic acid were higher in the 90% maturity melon than the 60% maturity group. Both acid synthesis and degradation processes determine the acid content of a fruit. This is in line with the results previously reported [35].

In our study, we applied RNA-seq analysis to meticulously examine the transcriptomic disparities between melons harvested at 60% and 90% maturity, spanning six distinct time-points during storage. The findings revealed pronounced transcriptomic differences between these two maturity stages, encompassing a total of 10,624 genes exhibiting statistically significant differential expression. Subsequent gene ontology (GO) enrichment analysis shed light on the functional implications of these differentially expressed genes. The up-regulated genes in melons harvested at 90% maturity displayed a greater association with metabolic, physiological, transport, and ion-binding processes. Conversely, the up-regulated genes in melons harvested at 60% maturity were notably enriched in GO categories such as catalytic activity and membrane localization. This comprehensive analysis underscores the substantial disparities in the expression patterns of up-regulated genes between melons harvested at 60% and 90% maturity, providing valuable insights into the molecular underpinnings of the observed differences in fruit quality during storage.

In addition to examining differential gene expression, our study delved into the role of alternative splicing, a process known to regulate multiple physiological processes during fruit ripening. Our findings revealed a substantial number of alternative splicing events occurring between melons harvested at 60% and 90% maturity, with the majority of these events involving skipped exons. Interestingly, a higher number of genes exhibited significant alternative splicing between the two maturity stages, aligning with the observed increase in differentially expressed genes over the storage period. These results collectively highlight a comprehensive molecular landscape, suggesting that not only gene expression, but also alternative splicing events, contribute significantly to the phenotypic divergence observed between melons harvested at 60% and 90% maturity during increased storage time. The intricate interplay between differentially expressed genes and alternative splicing events underscores the nuanced transcriptomic regulation that underlies the distinct profiles of metabolite accumulation in melon fruits at varying maturities.

## 5. Conclusions

In this study, we conducted a differential analysis of gene expression between 60% and 90% maturity stages of melon during different storage periods. Thousands of genes with differential expression were identified, indicating significant changes in transcript levels between these two ripening stages. We also found a significant role of alternative splicing (ASEs) in regulating physiological processes related to fruit ripening. Skipped exons were the most common ASEs, suggesting a crucial role for this mechanism in modifying genetic processes related to melon ripening. The results of the chemical analysis indicated significant differences between melons ripening at 60% and 90% maturity. Changes in sugar (sucrose, glucose, fructose) and organic acid (malic acid, citric acid, succinic acid) contents were observed, potentially influencing the taste and quality of the fruits. Taken together, these results suggest that differences in gene expression and alternative splicing may account for the observed phenotypic differences between 60% and 90% maturity melons.

## Figures and Tables

**Table 1:** Differentially expressed genes up-regulated relative to their expression at the two harvest time-points are associated with the sugar metabolism pathway.

Gene ID	Gene Name	Gene Description
MELO3C024383	CmCIN2	Cell wall invertase 2
MELO3C020357	CmSPS2	Sucrose-P synthase 2
MELO3C015469	CmBGAL3	β-galactosidase-like
MELO3C020357	CmSPS2	Sucrose-phosphate synthase
MELO3C001956	CmSUS-LIKE1	Sucrose synthase

## Data Availability

The sequencing data have been submitted to the NCBI SRA database (No. PRJNA914834).

## Supplementary Materials

The following supporting information can be downloaded at: https://www.mdpi.com/article/10.3390/genes15020149/s1, Table S1: Statistics of sequencing data across the 36 samples; Table S2: Read counts for each gene across the 36 samples; Table S3: FPKM for each gene across the 36 samples; Table S4: Differentially expressed genes of different time-points of storage between 60% and 90% melon maturity; Table S5: GO enrichment analyses of the differentially expressed genes of different time-point of storage between 60% and 90% melon maturity; Table S6: Alternative splicing events of different time-point of storage between 60% and 90% melon maturity; Figure S1: Protein–protein interactions for the DEGs using STRING database.
