# Supplementary material for: Time-Series Transcriptome of Cucumis melo Reveals Extensive Transcriptomic Differences with Different Maturity
Source: Genes (Basel). 2024 Jan 24;15(2):149. doi: 10.3390/genes15020149 (PMC10887994; doi:10.3390/genes15020149)
Supplement: Supplementary file 1 [file genes-15-00149-s001.zip › Figure S1.pdf]

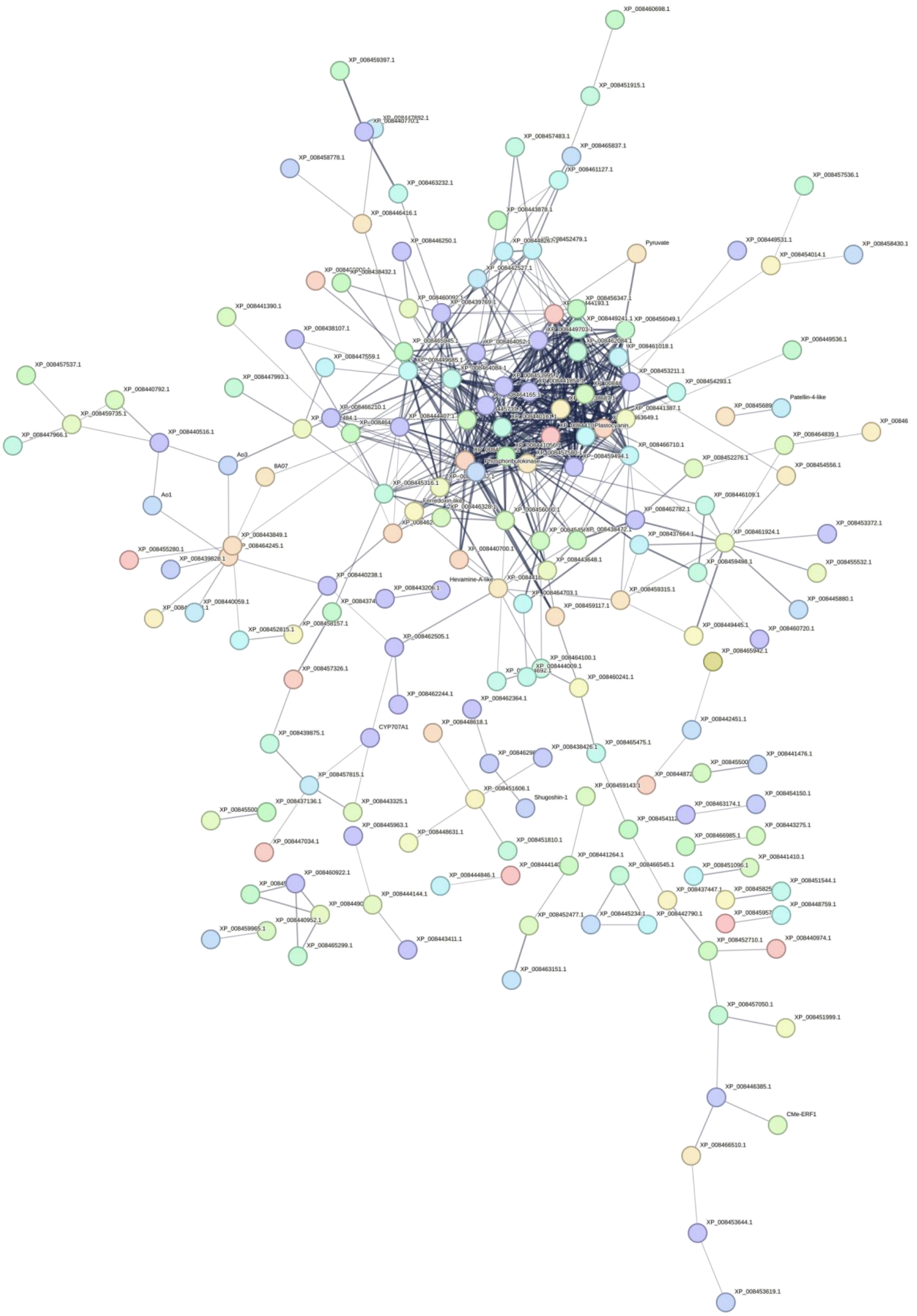

M33D_21_vs_M42D_day0.deseq2.up

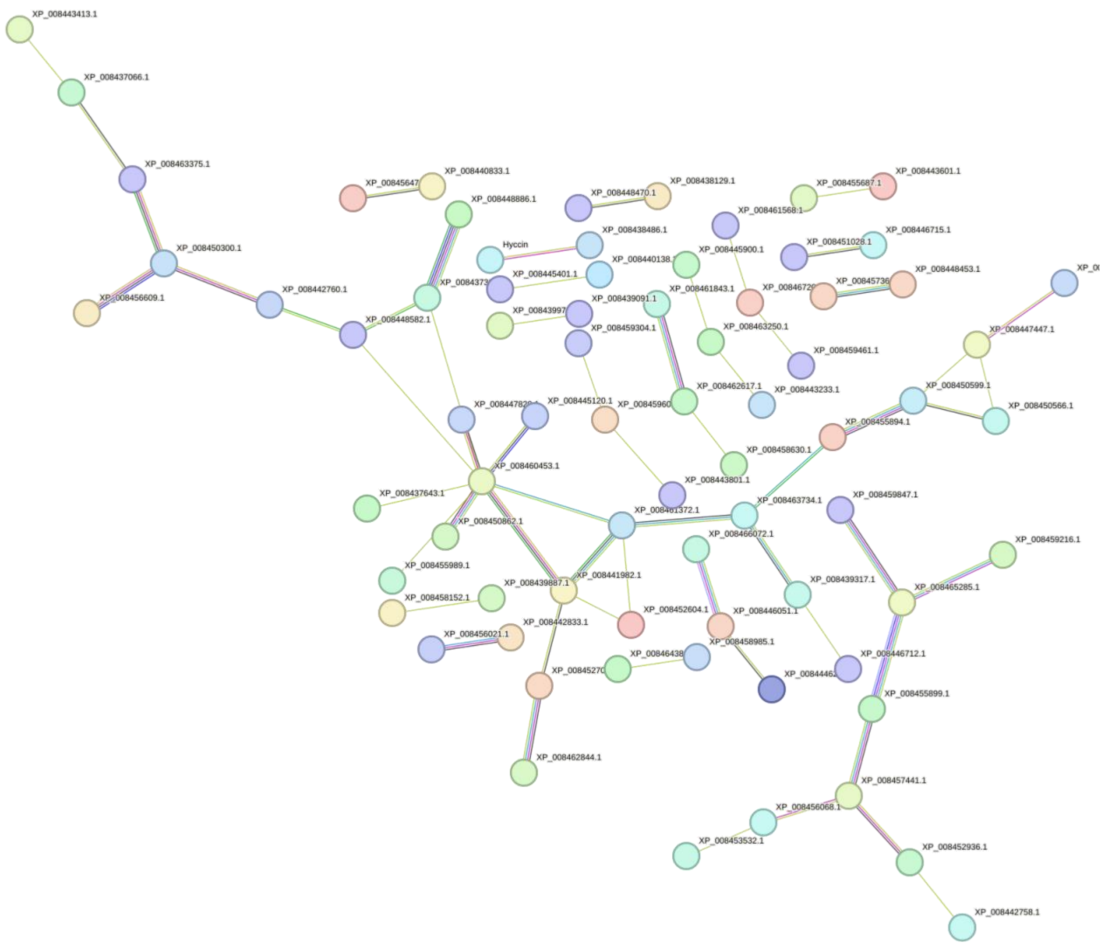

M33D_21_vs_M42D_day0.deseq2.down

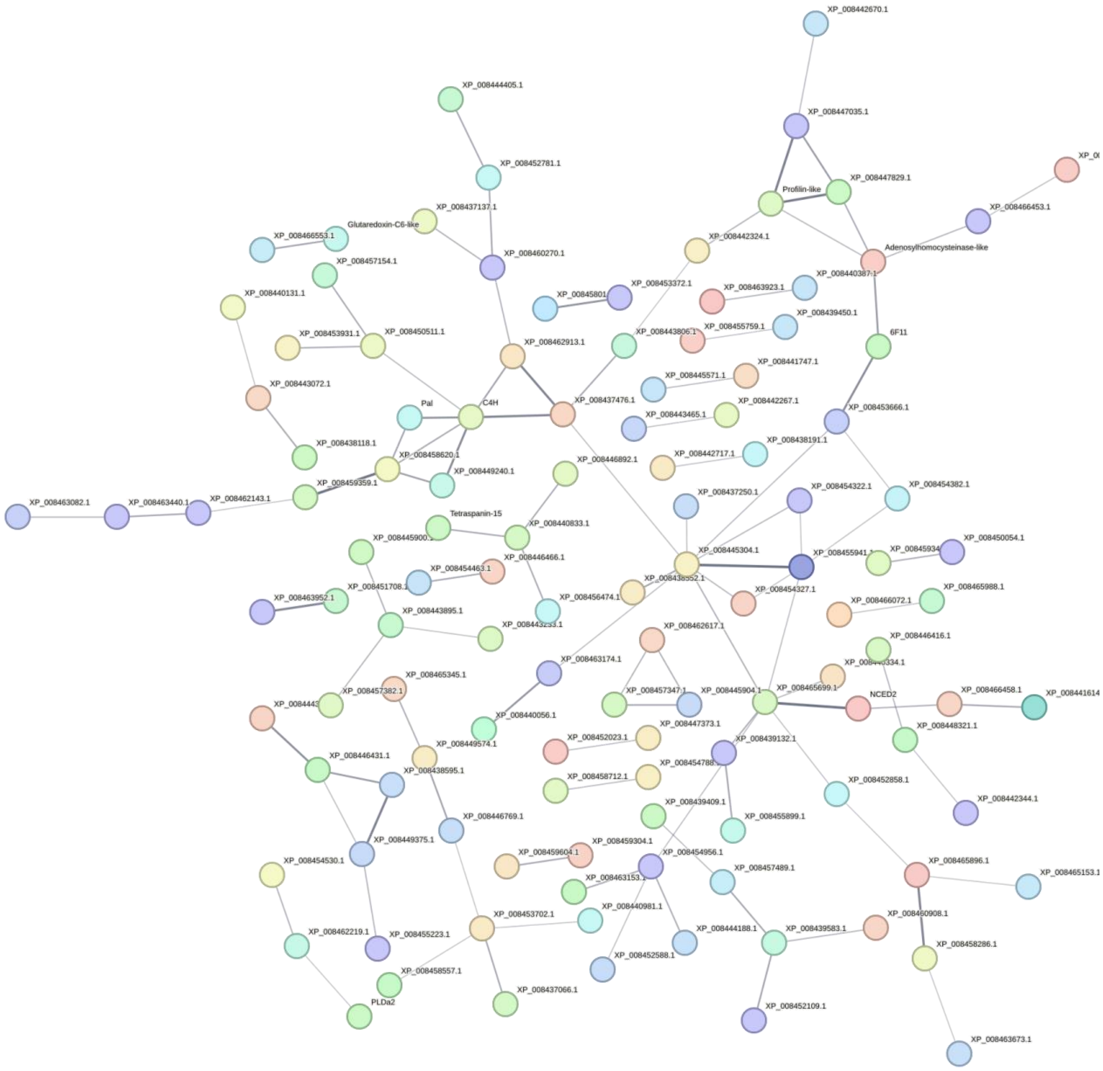

M33D_21_vs_M42D_day3.deseq2.up

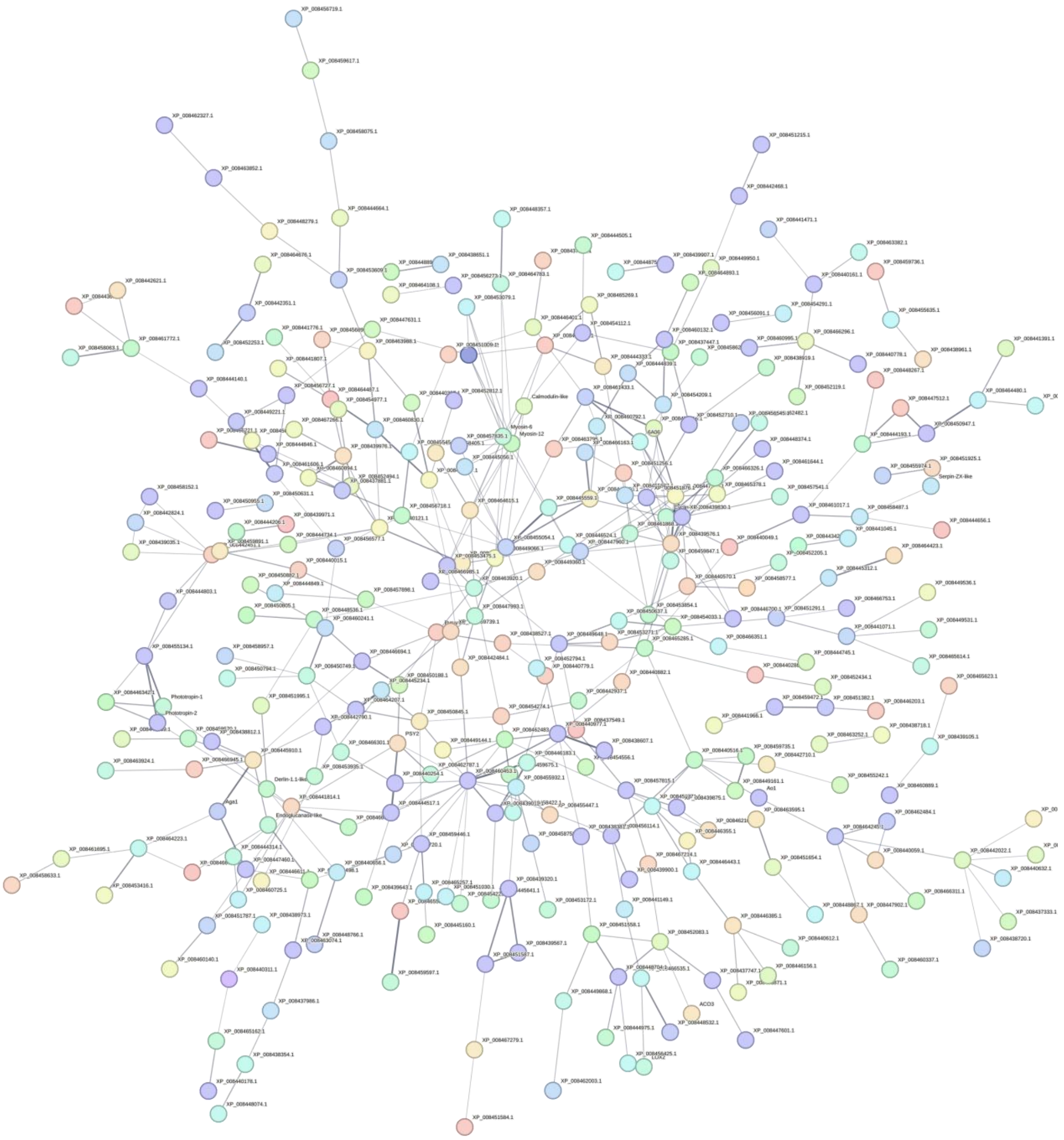

M33D_21_vs_M42D_day3.deseq2.down

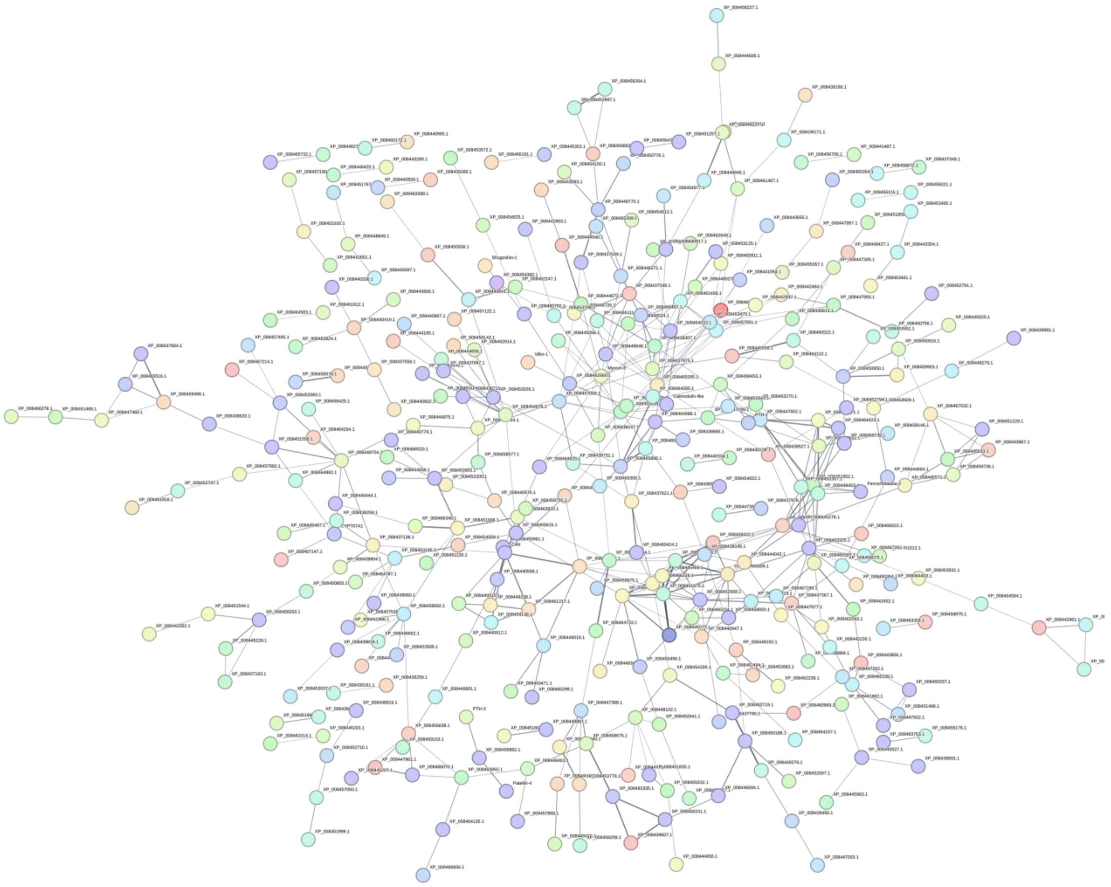

M33D_21_vs_M42D_day5.deseq2.up

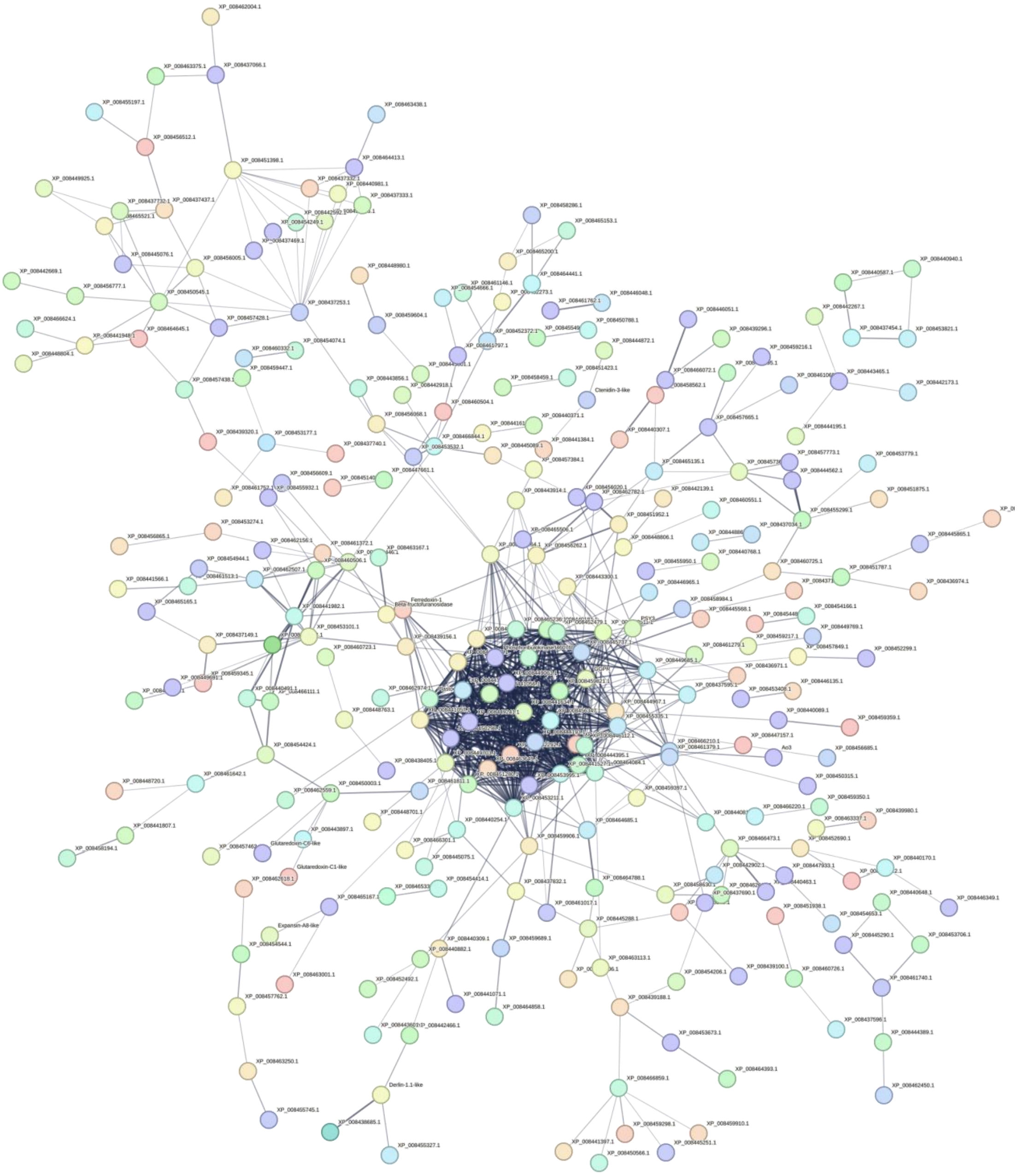

M33D_21_vs_M42D_day5.deseq2.down

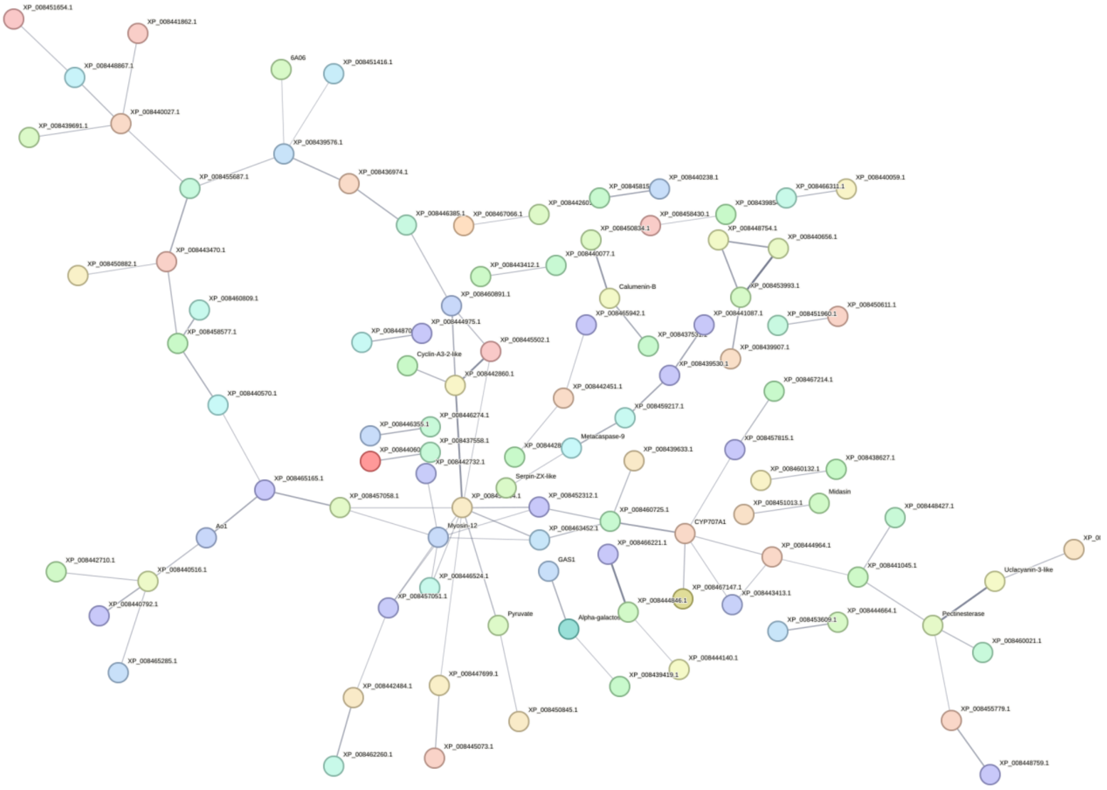

M33D_21_vs_M42D_day7.deseq2.up

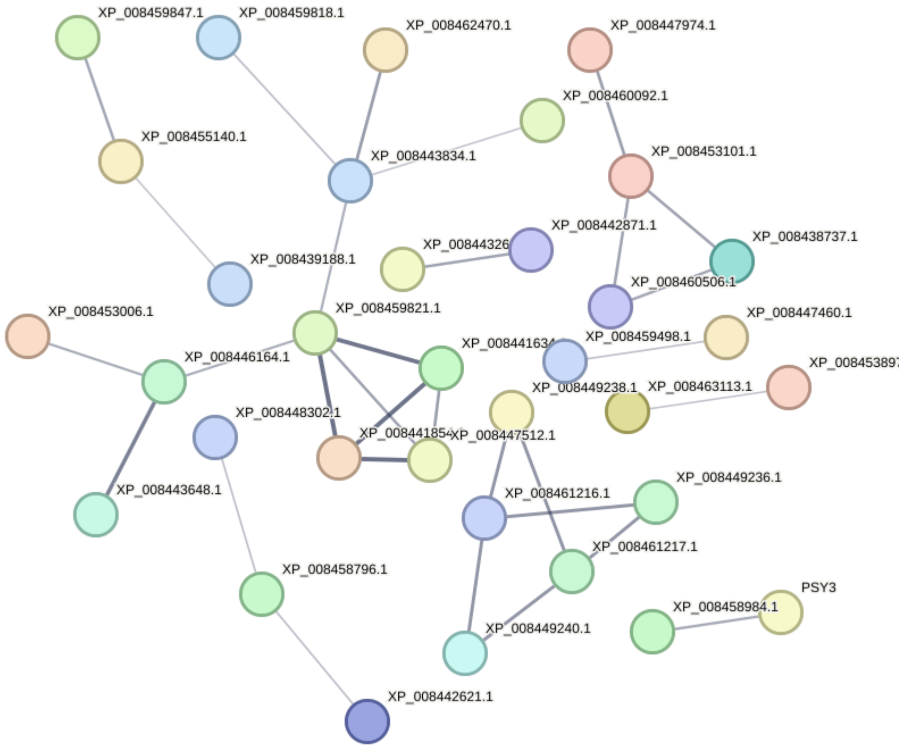

M33D_21_vs_M42D_day7.deseq2.down

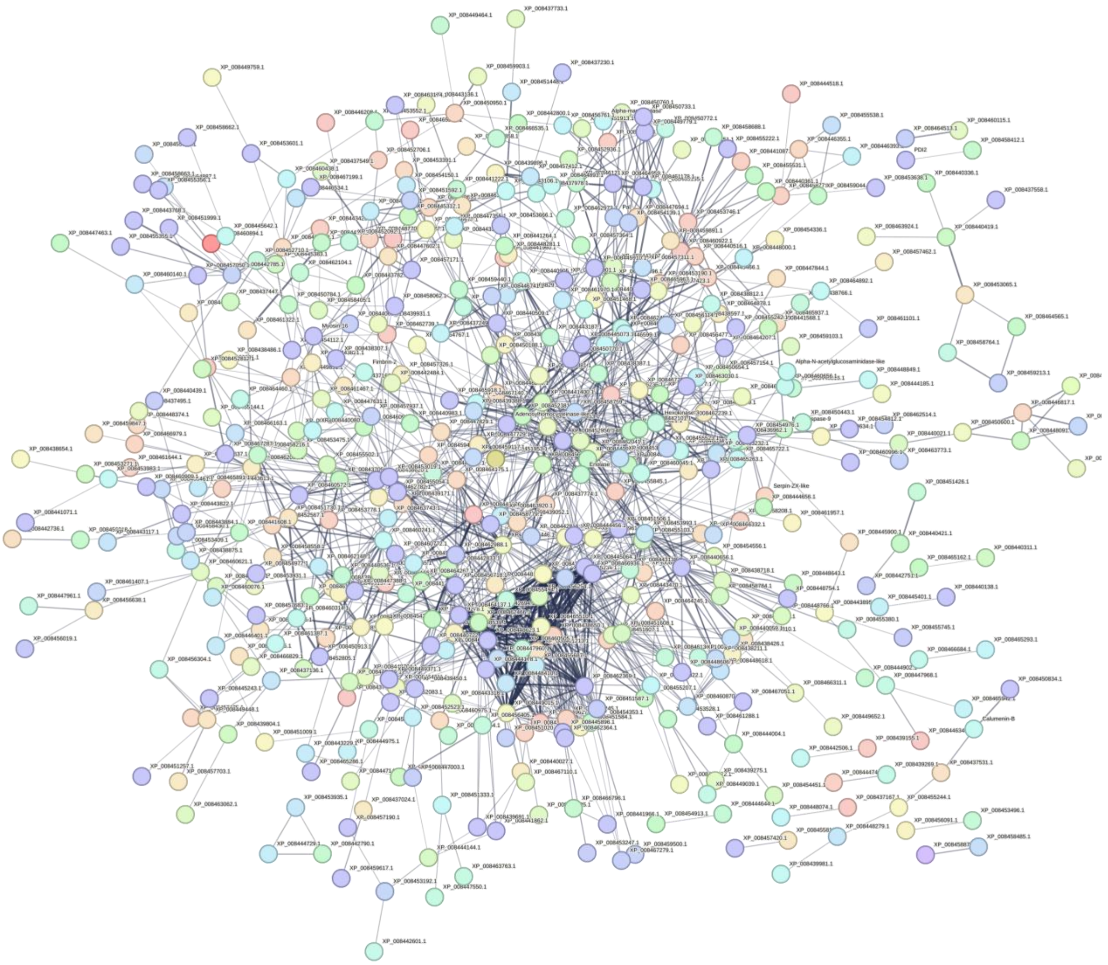

M33D_21_vs_M42D_day14.deseq2.up

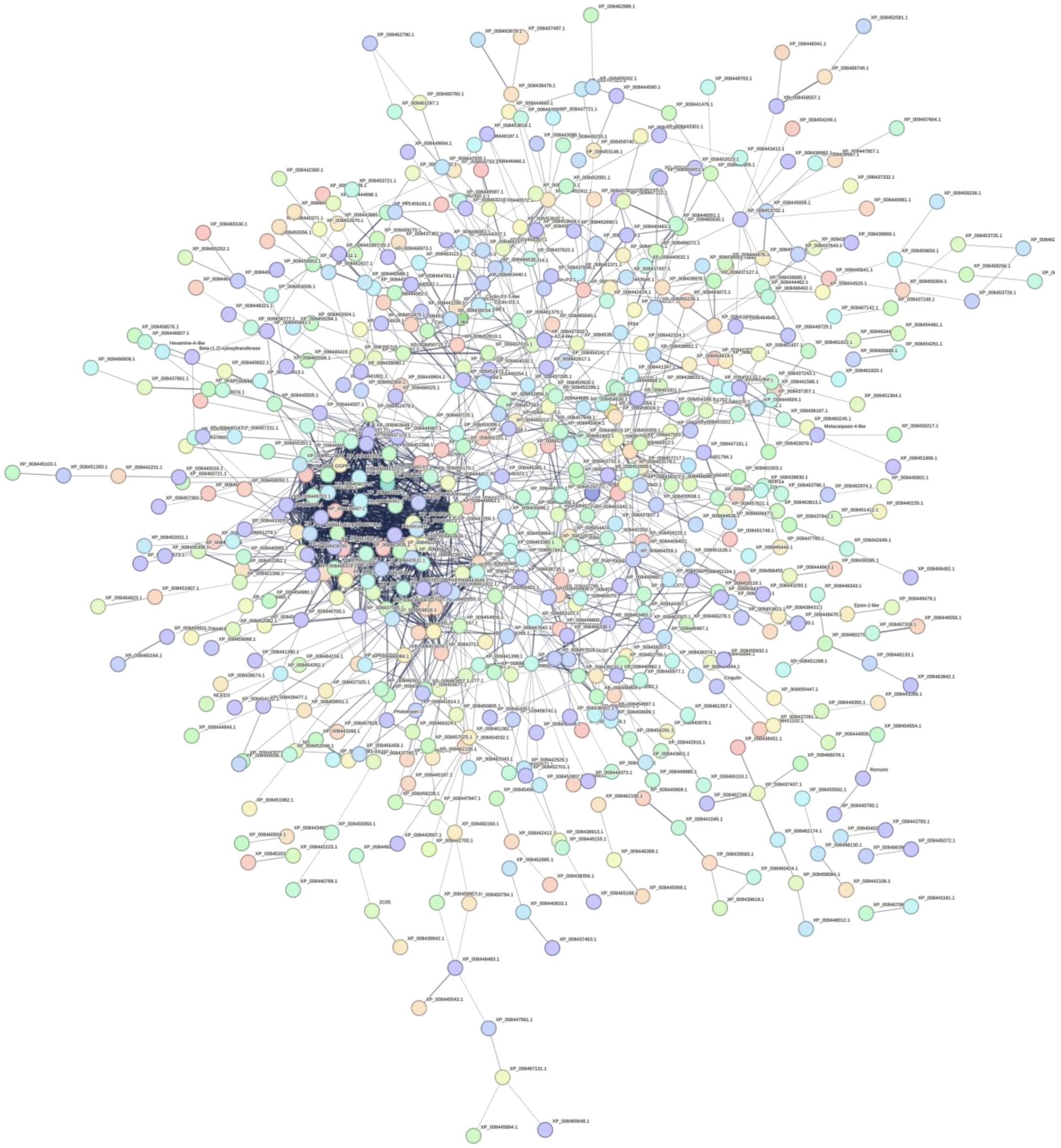

M33D_21_vs_M42D_day14.deseq2.down

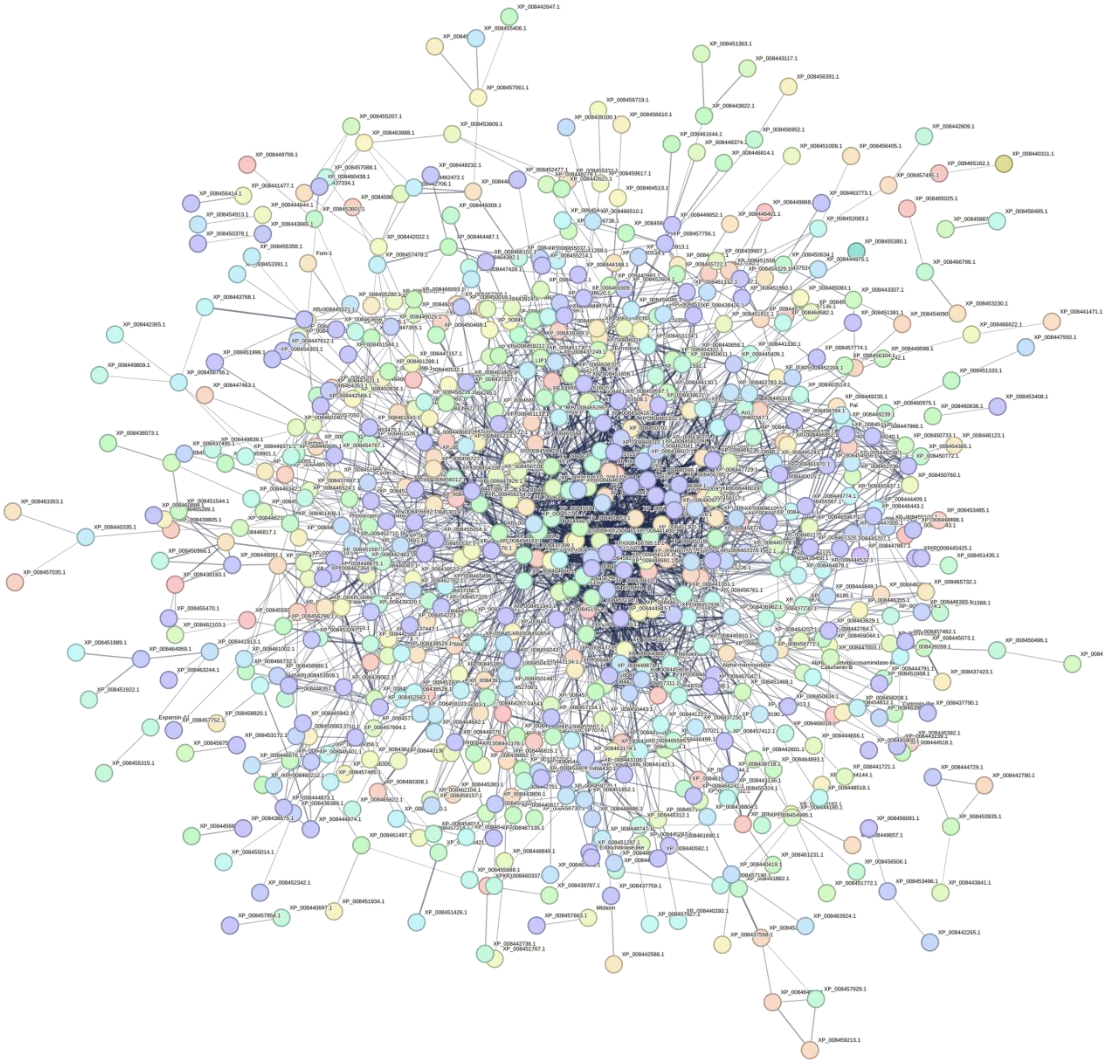

M33D_21_vs_M42D_day21.deseq2.up

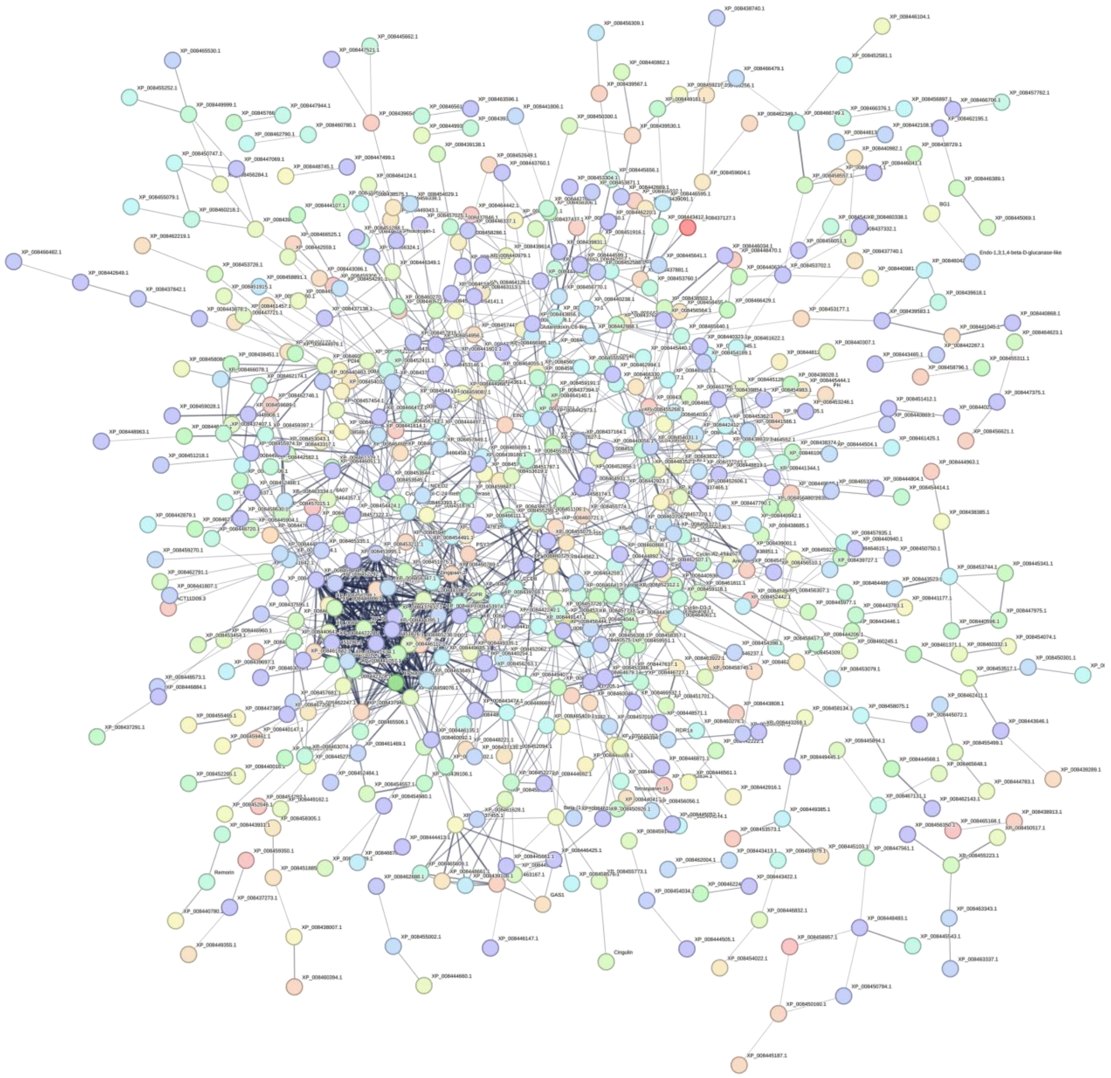

M33D_21_vs_M42D_day21.deseq2.down
